# The National Institutes of Health and guidance for reporting preclinical research

**DOI:** 10.1186/s12916-015-0284-9

**Published:** 2015-02-17

**Authors:** David Moher, Marc Avey, Gerd Antes, Douglas G Altman

**Affiliations:** Clinical Epidemiology Program, Ottawa Hospital Research Institute, Ottawa Hospital – General Campus, 501 Smyth Road, Room L1288, Ottawa, ON K1H 8L6 Canada; Department of Epidemiology and Community Medicine, University of Ottawa, Roger Guindon Hall, Room 3105, 451 Smyth Road, Ottawa, Ontario K1H 8M5 Canada; German Cochrane Centre, University Medical Center, Freiburg, Germany; Centre for Statistics in Medicine, Nuffield Department of Orthopaedics, Rheumatology & Musculoskeletal Sciences, University of Oxford, Botnar Research Centre Windmill Road, Oxford, OX3 7LD UK; UK EQUATOR Centre, University of Oxford, Oxford, UK

**Keywords:** Implementation, Preclinical research, Quality of reporting, Reporting guidelines

## Abstract

The quality of reporting clinical and preclinical research is not optimal. Reporting guidelines can help make reports of research more complete and transparent, thus increasing their value and making them more useful to all readers. Getting reporting guidelines into practice is complex and expensive, and involves several stakeholders, including prospective authors, peer reviewers, journal editors, guideline developers, and implementation scientists. Working together will help ensure their maximum uptake and penetration. We are all responsible for helping to ensure that all research is reported so completely that it is of value to everybody.

Please see related article: http://dx.doi.org/10.1186/s12916-015-0266-y

## Background

There is a large body of evidence indicating the very inadequate reporting of research, both clinical and preclinical. For example, in a survey of highly cited (>500 citations) animal studies from seven leading journals by impact factor (*Science*, *Nature*, *Cell*, *Nature Medicine*, *Nature Genetics*, *Nature Immunology*, and *Nature Biotechnology*) less than 20% of them described the randomization processes of the animals or blinding [1]. These findings have been consistent across preclinical research content areas (e.g., [2,3]).

To help increase the number of published papers that are completely and transparently reported, and thus reduce and/or eliminate the number of unusable publications, reporting guidelines have been developed since the early 1990s. There has been a proliferation in their numbers, particularly in the last decade. Reporting guidelines are popular; the EQUATOR Network’s library of reporting guidelines indicates more than 200 in existence, covering a wide variety of designs, data, populations, as well as clinical, preclinical, and other characteristics, with at least another score in development [4]. The Network is working to harmonize these activities and has also published advice on how to develop reporting guidelines [4], thus helping to ensure their rigorous development and usefulness. One reason for their popularity is that, when used appropriately by prospective authors, and endorsed and implemented optimally by journals, they have their intended effect, namely, of improving the completeness of research reports [5-7], thus increasing the value of research findings to clinicians, patients, and other interested readers.

## Recently published reporting guidelines

Another reporting guideline – CoBRA, indicating how to optimally cite bioresources – has been published in *BMC Medicine* [8]. At least two other reporting guidelines have been published this year already [9,10]. In 2013, the National Institutes of Health entered the fray, supported by the Nature Publishing Group and Science magazine, and proposed the Principles and Guidelines for Reporting Preclinical Research (NIHPG) [11] with a meeting involving 30 journal editors [12]. This triumvirate is an important and influential group. The NIHPG include the better performance of statistical analysis, transparency in reporting, data and material sharing, consideration of refutations, and consideration of establishing best practice guidelines, with a focus on authors providing complete information about the methods used in their studies to allow interested readers to replicate them. Within a short period of time the NIHPG have been endorsed by an impressive, and growing, number of journals, such as *Blood*, and groups, including the American Association for Cancer Research and the World Association of Medical Editors [13].

## Reporting preclinical research: how does NIHPG compare with existing guidelines?

In addition to the NIHPG, there are several other options [14-17] for prospective authors of preclinical research to report what they did and found, including the Animal Research: Reporting of In Vivo Experiments (ARRIVE) [18]. ARRIVE is a reporting guideline developed in partnership with the National Centre for the Replacement, Refinement and Reduction of Animals in Research [19]. The guidance was rigorously developed and followed a review of published animal studies that provided a rationale for moving forward. The development process also included a consensus meeting with a broad spectrum of stakeholders interested in animal research. At its core, the ARRIVE guidance is a 20-item checklist covering what authors should report in the Introduction, Methods, Results, and Discussion sections of their papers. For example, item 8 asks authors to report on the experimental animals used: “*Provide details of the animals used, including species, strain, sex, developmental stage (e.g. mean or median age plus age range) and weight*”. There is evidence that authors are not providing this information [20], despite it being essential if others are interested in replication [21].

The ARRIVE guidelines and NIHPG have different scope but similar objectives. The NIHPG is primarily focused on journal policy; for instance, the guidance recommends that journals have either no or generous limits on the length of their methods sections. In contrast, the ARRIVE guidelines are focused on reporting within the manuscript itself with its 20-item checklist, compared to the six ‘core’ reporting items found in the NIHPG. There is little difference between the six proposed items and their corresponding items (sub-items) in the ARRIVE checklist. Thus, the NIHPG items may be considered a minimum set of reporting items that are focused on the internal validity and statistics of a paper, whereas the ARRIVE guidelines are comprehensive and cover the entire paper from title to discussion. Indeed, the NIHPG even recommends following community standards such as the ARRIVE guidelines, suggesting that it is intended to co-exist with these other standards.

While the NIHPG and ARRIVE efforts to improve the reporting of preclinical research, are admirable, it is possible they might be seen as confusing, particularly to prospective authors. For example, in journals that endorse both ARRIVE (also endorsed by many journals – [22]) and NIHPG, authors might not know whether to use the NIHPG and/or ARRIVE and may elect to use neither, thus negating the efforts of both groups (in areas of overlap, such as statistics, should authors choose one guidance over another one and, if so, which one?). Indeed, a similar situation occurred during the developing of the CONSORT Statement for reporting randomized trials. The CONSORT guidance was produced about 6 months before the Asilomar guidance. Dr. Drummond Rennie, then deputy editor of *JAMA*, recommended the two groups work together to produce a single guidance thus providing more clarity and direction for prospective authors. Similarly, the CONSORT group wanted to incorporate the TIDieR guidance for describing interventions [23] into the CONSORT checklist. Members of both groups discussed how best to do this and provided some guidance on this for prospective authors [23].

While the NIHPG guidance appears to have face validity and support, a more pressing question is whether it will have its intended effect, namely, improving the completeness and transparency when reporting preclinical research, as well as facilitating the likelihood of reproducible studies being done. There is likely little merit in editors asking authors to use the guidance – the intervention – if it has only a minimal effect on relevant outcomes such as better reporting and enabling others to adequately replicate methods. Unfortunately, an assessment of the benefits (and harms) of reporting guidelines has not been a top priority of guideline developers [24], to date. We hope the NIHPG developers will actively plan to conduct an evaluation of their guidance and encourage others to do likewise. Attention to relevant outcomes will be important to consider. Evaluations assessing adherence to reporting guidelines (i.e., in addition to endorsement) may provide a more meaningful insight into its impact on completeness of reporting when used at different stages of the editorial process. One such recently performed evaluation [25] incorporated these concepts by comparing the use and non-use of reporting guidelines during peer review on author-revised manuscript quality.

Having produced the NIHPG – an intervention to help improve the transparency and better reporting of preclinical research – it is important to consider its endorsement and implementation. More generally, are journals using similar explicit language regarding the endorsement of either guidance? Our experiences are that there is a wide variability in the language of endorsement and that this is confusing to prospective authors, thus potentially reducing the intended effectiveness of the guidance. Guideline developers are starting to provide greater guidance to help journal editors achieve a more standard and successful endorsement strategy [9].

## Implementing reporting guidelines

While endorsement is an important step, how journals implement reporting guidelines is a critical factor. There is wide variability in how journals implement reporting guidelines [26] possibly because guideline developers have not focused on how to do this until recently [27,28]. If editorial procedures are not consistently available online it will be difficult to assess journal implementation of NIHPG endorsement (that is, verification by the journals’ editorial team of author adherence to it). Editors may want to develop explicit statements about their journals’ endorsement of NIHPG, and other reporting guidelines, in their ‘Instructions to Authors’, and optimally to recommend submission of relevant checklists at the time of manuscript submission. To further implementation practices, editors may also want to recommend peer reviewers use reporting guidelines during their manuscript review assessment [29]. Such active implementation policies by journals will lead to more complete, clear, transparent, and reproducible publications, ultimately increasing the usability and value of preclinical research reports.

Well-developed reporting guidelines can improve the reporting and usefulness of all research. If studies are reported with enough detail for the findings to be implemented in practice, they are more helpful to health care providers, policy makers, and patients for making decisions. Transparent reporting also allows decision makers to judge the internal validity and applicability of the research, and enables others to reproduce the findings. Currently we do not know which reporting guidelines are most helpful in achieving this goal because of the variability in their development; this was a discussion theme at a recent EQUATOR Network meeting [30]. The development of a tool to assess the robustness (and other features) of reporting guidelines will help all stakeholders, particularly research funders and journal editors, to make decisions on which reporting guidelines to focus their endorsement and implementation efforts. Similarly, robust guidelines are likely the ones for prospective authors to spend their time using.

Getting reporting guidelines into practice is complex and expensive, and involves many players, including prospective authors, peer reviewers, journal editors, guideline developers, and implementation scientists. Working together will help ensure their maximum uptake and penetration. Technology is also likely to facilitate implementation of reporting guidelines, such as software to help prospective authors ensure they remember to report on every aspect of the research they are reporting, and journal editors to assess the degree of compliance to guidelines they endorse. Unfortunately, to date, there has been very limited funding available to develop and help maximize the potential of reporting guideline uptake. Perhaps having the NIH involved in reporting guideline efforts will encourage them and others to commit more resources to these efforts. We are all responsible for helping to ensure that all research is reported so completely that it is of value to everybody.

## Abbreviations

ARRIVE: Animal Research: Reporting of In Vivo Experiments
NIHPG: National Institutes of Health Principles and Guidelines for Reporting Preclinical Research
