# Erratum: the National Institutes of Health and guidance for reporting preclinical research

**DOI:** 10.1186/s12916-015-0321-8

**Published:** 2015-04-10

**Authors:** David Moher, Marc Avey, Gerd Antes, Douglas G Altman

**Affiliations:** Clinical Epidemiology Program, Ottawa Hospital Research Institute, Ottawa, Canada; Department of Epidemiology and Community Medicine, University of Ottawa, Ottawa, Canada; German Cochrane Centre, University Medical Center, Freiburg, Germany; Centre for Statistics in Medicine, University of Oxford, Oxford, UK

## Abstract

This is an Erratum to BMC Medicine 2015, 13:34, highlighting a corrected references list.

Please see related article: http://www.biomedcentral.com/1741-7015/13/34

## Erratum

### Authors’ corrected note

Two days after the publication of our paper [1], a reader brought to our attention some problems with our references. The erratum corrects the reference list.

### Corrected text

Reference [4] before, “thus helping to ensure…” should be - 5. Moher D, Schulz KF, Simera I, Altman DG.Guidance for Developers of Health Research Reporting Guidelines. PLoS Med. 2010;7(2): e1000217.

References [5-7] should be - 6. Turner L, Shamseer L, Altman DG, Weeks L, Peters J, Kober T, Diaz S, Schulz KF, Plint AC, Moher D. Consolidated standards of reporting trials (CONSORT) and the completeness of reporting of randomized controlled trials (RCTs) published in medical journals. Cochrane Database Syst Rev. 2012;11:MR000030.

7. Smidt N, Rutjes AW, van der Windt DA, Ostelo RW, Bossuyt PM, Reitsma JB, Bouter LM, de Vet HC. The quality of diagnostic accuracy studies since the STARD statement: has it improved? Neurology. 2006;67:792–7.

8. Tunis AS, McInnes MD, Hanna R, Esmail K. Association of study quality with completeness of reporting: have completeness of reporting and quality of systematic reviews and meta-analyses in major radiology journals changed since publication of the PRISMA statement? Radiology. 2013;269:413–26.

Reference [8] should be - 9. Bravo E, Calzolari A, De Castro P, Mabile L, Napolitani F, Rossi AM, Cambon-Thomsen A. Developing a guideline to standardize the citation of bioresources in journal article (CoBRA). BMC Medicine 2015.

References [9-10] should be - 10. Moher D, Shamseer L, Clarke M, Ghersi D, Liberati A, Petticrew M, Shekelle P, Stewart LA. Preferred reporting items for systematic review and meta-analysis protocols (PRISMA-P) 2015 statement. Syst Rev. 2015;4:1.

11. Collins GS, Reitsma JB, Altman DG, Moons KG. Transparent reporting of a multivariable prediction model for individual prognosis or diagnosis (TRIPOD): The TRIPOD statement. Ann Intern Med. 2015;162(1):55–63.

Reference [11] should be - 12. http://www.nih.gov/about/reporting-preclinical-research.htm.

Reference [12] should be - 13. Nature editorial. Announcement: Reducing our irreproducibility. Nature 496, 398.

Reference [13] should be - 14. http://www.nih.gov/about/endorsing-jounals.htm.

References [14-17] should be - 15. Hooijmans CR, Leenaars M, Ritskes-Hoitinga M. A gold standard publication checklist to improve the quality of animal studies, to fully integrate the Three Rs, and to make systematic reviews more feasible. Altern Lab Anim. 2010;38:167–82.

16. Institute for Laboratory Animal Research. Guidance for the Description of Animal Research in Scientific Publications. 1–31 (The National Academies Press, Washington DC, 2011).

17. Landis SC, Amara SG, Asadullah K, Austin CP, Blumenstein R, Bradley EW, Crystal RG, Darnell RB, Ferrante RJ, Fillit H, Finkelstein R, Fisher M, Gendelman HE, Golub RM, Goudreau JL, Gross RA, Gubitz AK, Hesterlee SE, Howells DW, Huguenard J, Kelner K, Koroshetz W, Krainc D, Lazic SE, Levine MS, Macleod MR, McCall JM, Moxley RT 3rd, Narasimhan K, Noble LJ, et al. A call for transparent reporting to optimize the predictive value of preclinical research. Nature. 2012;490,187–91.

18. Nature. Reporting Checklist For Life Sciences Articles [Internet]. 2013. http://www.nature.com/authors/policies/checklist.pdf Last accessed on: 01-15-2015.

Reference [18] should be - 19. Kilkenny C, Browne WJ, Cuthill IC, Emerson M, Altman, DG. The ARRIVE guidelines Animal Research: Reporting In Vivo Experiments. PLoS Biol. 2010. 8, e1000412.

Reference [19] should be - 20. http://www.nc3rs.org.uk/.

Reference [20] should be - 21. Baker D, Lidster K, Sottomayor A, Amor S. Two years later: journals are not yet enforcing the ARRIVE guidelines on reporting standards for pre-clinical animal studies. PLoS Biol 2013;11:e1001756.

Reference [21] should be - 22. Collins FS, Tabak LA. NIH plans to enhance reproducibility. Nature 2014;505:612–613.

Reference [22] should be - 23. https://www.nc3rs.org.uk/arrive-animal-research-reporting-vivo-experiments.

Reference [23] should be - 24. Hoffmann TC, Glasziou PP, Boutron I, Milne R, Perera R, Moher D, Altman DG, Barbour V, Macdonald H, Johnston M, Lamb SE, Dixon-Woods M, McCulloch P, Wyatt JC, Chan AW, Michie S. Better reporting of interventions: template for intervention description and replication (TIDieR) checklist and guide. BMJ. 2014 Mar 7;348:g1687.

Reference [24] should be - 25. Stevens A, Shamseer L, Weinstein E, Yazdi F, Turner L, Thielman J, Altman DG, Hirst A, Hoey J, Palepu A, Schulz KF, Moher D. Relation of completeness of reporting of health research to journals’ endorsement of reporting guidelines: systematic review. BMJ. 2014 Jun 25;348:g3804.

Reference [25] should be - 26. Cobo E, Cortés J, Ribera JM, Cardellach F, Selva-O'Callaghan A, Kostov B, García L, Cirugeda L, Altman DG, González JA, Sànchez JA, Miras F, Urrutia A, Fonollosa V, Rey-Joly C, Vilardell M. Effect of using reporting guidelines during peer review on quality of final manuscripts submitted to a biomedical journal: masked randomised trial. BMJ. 2011 Nov 22;343:d6783.

Reference [26] should be - 27. Hirst A, Altman DG: Are peer reviewers encouraged to use reporting guidelines? A survey of 116 health research journals. PLoS ONE 2012, 7(4):e35621.

Reference [27,28] should be - 28. Michie S, Johnston M. Theories and techniques of behaviour change: Developing a cumulative science of behaviour change. Health Psychol Rev. 2012;6(1):1–6.

29. French SD, Green SE, O’Connor DA, McKenzie JE, Francis JJ, Michie S, Buchbinder R, Schanttner P, Spike N, Grimshaw JM. Developing theory-informed behaviour change interventions to implement evidence into practice: a systematic approach using the Theoretical Domains Framework. Implement Sci. 2012;7:38.

Reference [29] should be – 30. http://www.equator-network.org/library/.

Reference [30] should be - 31. http://www.equator-network.org/2014/11/25/the-first-equator-reporting-guideline-development-meeting-18-20-november-2014-oxford/.
